# The Cost-Effectiveness of Hepatitis C Virus Screening Strategies among Recently Arrived Migrants in the Netherlands

**DOI:** 10.3390/ijerph17176091

**Published:** 2020-08-21

**Authors:** Mohamed N.M.T. Al Khayat, Job F.H. Eijsink, Maarten J. Postma, Jan C. Wilschut, Marinus van Hulst

**Affiliations:** 1 Department of Health Sciences, University of Groningen, University Medical Center Groningen, 9712 CP Groningen, The Netherlands; j.f.h.eijsink@isala.nl (J.F.H.E.); m.j.postma@rug.nl (M.J.P.); r.hulst@mzh.nl (M.v.H.); 2 Department of Clinical Pharmacy, Isala Hospital Zwolle, 8025 BT Zwolle, The Netherlands; 3 Department of Economics, Faculty of Economics & Business, University of Groningen, Econometrics & Finance, 9712 CP Groningen, The Netherlands; 4 Department of Medical Microbiology, University of Groningen, University Medical Center Groningen, 9712 CP Groningen, The Netherlands; jcwilschut@gmail.com; 5 Unit of Pharmacotherapy, Epidemiology & Economics, Department of Pharmacy, University of Groningen, 9712 CP Groningen, The Netherlands; 6 Department of Clinical Pharmacy, Martini Hospital Groningen, 9728 NT Groningen, The Netherlands

**Keywords:** hepatitis C, screening, migrants, cost-effectiveness, budget impact

## Abstract

Objective: We aimed to assess the cost-effectiveness of hepatitis C virus (HCV) screening strategies among recently arrived migrants in the Netherlands. Methods: A Markov model was used to estimate the health effects and costs of HCV screening from the healthcare perspective. A cohort of 50,000 recently arrived migrants was used. In this cohort, three HCV screening strategies were evaluated: (i) no screening, (ii) screening of migrants from HCV-endemic countries and (iii) screening of all migrants. Results: Strategy (ii) screening of migrants from HCV-endemic countries compared to strategy (i) no screening, yielded an incremental cost-effectiveness ratio (ICER) of €971 per quality-adjusted life-years (QALYs) gained. Strategy (iii) screening of all migrants compared with strategy (ii) screening of migrants from HCV-endemic countries yielded an ICER of €1005 per QALY gained. The budget impact of strategy (ii) screening of migrants from HCV-endemic countries and strategy (iii) screening of all migrants was €13,752,039 and €20,786,683, respectively. Conclusion: HCV screening is cost-effective. However, the budget impact may have a strong influence on decision making.

## 1. Introduction

Hepatitis C virus (HCV) prevalence in the Netherlands ranges between 0.1% and 0.4%, and is higher in specific risk populations, such as migrants [1,2]. Specifically, an HCV prevalence of 0.7% up to 2.3% was estimated among first-generation migrants in the Netherlands [3,4]. The total number of first-generation migrants from Middle Eastern countries who were living in the Netherlands in 2016 was 1,148,449, representing about 7% of the total population [2,5]. HCV prevalence is region-specific, with, for example, relatively high rates in the Middle East. The Arabic spring eventually led to a wave of violence and war, resulting in an influx of migrants from the Middle East into Europe [6]. Between 2004 and 2015, annually, 90,000 to 182,000 immigrants arrived in the Netherlands, many from the Middle East. Approximately 30% of recently arrived immigrants were born in countries with an HCV prevalence of 2% and higher. Recently arrived migrants are defined as migrants who arrived in the Netherlands during the last year. HCV prevalence and the related burden of hepatitis C in the Netherlands is likely to increase over the next decades as a result of the growing number of people immigrating from HCV-endemic countries [2,7].

Treatment for HCV has improved significantly since the introduction of new direct-acting antivirals (DAAs), which present options for cure. DAAs have rapidly improved the sustained virologic response (SVR) rates for HCV treatment [8,9]. While previously only 50% of those infected with HCV could be treated successfully [10], the new DAA treatment regimens can potentially achieve SVR in over 90% of patients [8,9]. With these highly effective treatments for HCV, implementation of an HCV screening program among migrants might be warranted. Indeed, an HCV screening program in combination with treatment would avoid HCV-related liver disease, particularly by detecting HCV patients in an asymptomatic or early stage of the disease. However, the cost-effectiveness of such an HCV screening program is unknown.

The Health Council in the Netherlands has explicitly recommended a cost-effectiveness and public-health impact analysis for HCV screening among migrants [1]. In this study, we conducted such a cost-effectiveness analysis of HCV screening among migrants in the Netherlands. The estimated cost-effectiveness as well as budget impact of such an HCV screening program is expected to help policymakers to decide whether screening should be implemented and which groups could potentially be screened cost-effectively in the context of different strategies.

## 2. Materials and Methods

### 2.1. Course of Disease

HCV causes acute and chronic infection. Around 30% (15–45%) of infected patients spontaneously clear the virus within 6 months of infection without any treatment. Therefore, 70% (55–85%) of patients will develop chronic HCV infection [11]. Liver cirrhosis will develop in 15% to 30% of patients with chronic HCV infection. In cirrhotic patients, the risk of hepatocellular carcinoma (HCC) is 2% to 4% annually [12,13]. Patients are classified in METAVIR scores after diagnosis of HCV infection. The METAVIR score grades the degree of fibrosis on a 5-point scale from 0 to 4. F0–F4 stages are defined as: no fibrosis (F0), portal fibrosis without septa (F1), portal fibrosis with few septa (F2), numerous septa without fibrosis (F3) and compensated cirrhosis (F4). Without screening, the distribution of HCV-infected patients over stages F0, F1, F2, F3 and F4 is 17%, 35%, 22%, 14% and 12%, respectively [14]. Chronic HCV infection is often asymptomatic and only 25% to 30% of infected persons seek medical attention for symptoms attributable to HCV infection [15]. Since only 25% to 30% of individuals with hepatitis C are diagnosed, we estimated that screening will identify the asymptomatic patients. Studies show that asymptomatic HCV patients are mostly in stage F0 in the first years after infection [16]. Therefore, among migrants screened for HCV, we estimated that the distribution of HCV-positive tested migrants over stages F0, F1, F2, F3 and F4 is 89%, 8%, 2%, 0.8% and 0.2%, respectively [14]. Therefore, an HCV screening program in combination with treatment avoids HCV-related liver diseases by detecting HCV patients in an asymptomatic or early stage of the disease.

### 2.2. Model

**Figure 1:**
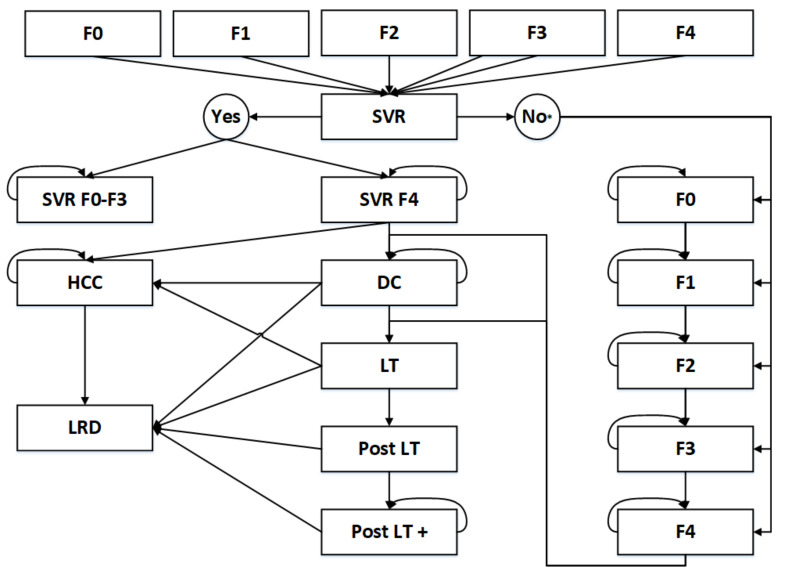
Markov model for hepatitis C virus (HCV). METAVIR score: F0, F1, F2, F3, F4; SVR: Sustained Virologic Response; HCC: hepatocellular carcinoma; DC: decompensated cirrhosis; LT: liver transplantation; LRD: liver-related death. * In case of treatment failure, patients will be in the same METAVIR state after the treatment.

To estimate the cost-effectiveness of HCV screening in the Netherlands, we modeled chronic hepatitis C. Specifically, a Markov model was developed in Microsoft Excel 2016 (Figure 1). We assumed that migrants remain in the Netherlands for the rest of their lives. Therefore, Dutch life tables were applied for life expectancy and we used a corresponding lifetime horizon. In the Markov model, we used an annual cycle to model the progression of HCV.

In the model, HCV patients can be treated with new DAA once HCV is detected by screening or diagnosed by symptoms. Patients are classified in METAVIR stages after diagnosis of HCV infection. In the model, if patients do not reach an SVR after DAA treatment, they may progress from METAVIR F0, F1, F2, F3, F4 to decompensated cirrhosis (DC), HCC, liver-related death (LRD), liver transplantation (LT) and post-liver transplantation care (LT+). The parameters for the transition state probabilities were extracted from the literature and have been applied in the most recent studies to model HCV in the Netherlands [17,18,19,20,21]. The cost parameters were extracted from the healthcare institute in the Netherlands [22]. All parameters used in the hepatitis C model are presented in Table S1.

### 2.3. Epidemiology of HCV among Middle Eastern Migrants in The Netherlands

HCV prevalence among first-generation, recently arrived migrants from countries in the Middle East varies between 0.7% and 2.3% [3,4,23]. The country of origin, HCV prevalence and age distribution of recently arrived migrants in the Netherlands are presented in Table 1 and Table 2. Notably, five countries in the Middle East represent the countries with the highest migration to the Netherlands. Indeed, 51% of migrants in the Netherlands are Syrians with an HCV prevalence of 1.0% [24]. In our study, the HCV prevalence in the cohort of all migrants is estimated at 1.5%, based on the weighted average HCV prevalence among all recently arrived migrants. For the cohort of migrants from endemic countries, we estimated the HCV prevalence at 2%.

### 2.4. HCV Screening and Treatment

HCV screening in this study consists of three different tests. The first test is a serological test, which determines the presence of antibodies against HCV. This test identifies a previous infection with HCV. The second test is a polymerase chain reaction (PCR) test, which determines the presence of viral RNA. A negative PCR test in combination with a positive serological test identifies those individuals who cleared the virus spontaneously after HCV infection. The sensitivity and specificity of the PCR test are 100% [25]. The third test is a fibroscan test to determine the METAVIR score (F0–F4). The METAVIR score, as indicated above, shows the severity of disease and the progression from liver fibrosis to cirrhosis [26]. The genotype testing is not included in our evaluation, because most of the new DAAs cover all HCV genotypes. In the model, HCV patients with a positive PCR test are treated with DAAs. We assumed that recently arrived migrants had no co-infection with hepatitis B virus (HBV) or human immunodeficiency virus (HIV) [27]. Since 2014, DAA treatment has reached SVR levels of 90% in the F4 METAVIR group and up to 95% in the F0–F3 METAVIR groups for all viral genotypes [8,9,28]. In the model, we assumed 95% SVR for patients in F0–F3 after 12 weeks of treatment and 90% SVR for patients in F4 after 24 weeks of treatment [29,30]. We assumed that there is no further transmission among migrants. The HCV patients in this group are mostly infected due to poor healthcare practices in their home countries. Blood–blood transmission due to poor healthcare practices is extremely unlikely in the Netherlands. In our model, patients with compensated cirrhosis (F4) are at risk for developing DC and HCC, even after reaching an SVR state [31]. We did not model treatment uptake because uptake in the Netherlands in high due to unrestricted access to DAA since 2015 [32].

Given the SVR rates stated above, 5% of F0–F3 and 10% of F4 patients would not reach a SVR and would be able to develop HCV-related liver disease after treatment failure [33]. The cost of the treatment was based on the average cost of DAA pan genotypic regimes [22,29,30,34,35].

### 2.5. Health Outcomes

Quality-adjusted life-years (QALYs) were used as a measure of health outcomes. Specifically, in our model, QALYs were estimated by multiplying the utility value associated with a given state of health (quality of life) by the years lived in that state [14,36,37]. Patients achieving SVR remain in the last disease state with a utility of 0.05 awarded for successful treatment [38,39].

### 2.6. Screening and Treatment Strategies

The cost-effectiveness and budget impact of three strategies for HCV screening and subsequent treatment in recently arrived migrants were evaluated: (i) no screening, (ii) screening of migrants from HCV-endemic countries and (iii) screening of all migrants. The no screening strategy reflects the current practice. The screening of migrants from HCV-endemic countries strategy combines HCV screening of migrants from HCV-endemic countries with an HCV prevalence of >2% and no screening of migrants from non-HCV-endemic countries. In this strategy, an HCV prevalence of 2% was used in the base case, as a conservative strategy. All recently arrived migrants are screened for HCV in the screening of all migrants strategy. The HCV prevalence used in this strategy is estimated to be 1.5% based on the weighted average HCV prevalence among all migrants.

The three strategies were evaluated in a cohort of recently arrived migrants in the Netherlands. This cohort concerns migrants who arrived in the Netherlands during the year before the start of the screening program. The migrants would be screened during their stay in the Dutch Asylum Seekers Centre. The number of arrived migrants during one year in the Netherlands was collected from the Central Bureau of Statistics in the Netherlands in 2016 [2].

### 2.7. Cost-Effectiveness and Budget-Impact Analyses

The health effects and costs of HCV screening and treatment were estimated from the healthcare perspective. The results of the cost-effectiveness analyses are expressed in terms of the Incremental Cost-Effectiveness Ratio (ICER). The ICER represents the ratio of the incremental costs over the QALYs gained [40].

We used an annual discounting rate of 4% for costs and 1.5% for QALYs according to Dutch guidelines [41]. We used the informal Dutch willingness-to-pay (WTP) threshold of €20,000 per QALY gained for screening programs in order to determine if screening for chronic HCV infection would be cost-effective [22].

The budget-impact analysis gives a perspective on future HCV-related costs. For the budget-impact analysis, we included direct medical costs, costs of HCV treatment and costs of screening, in the first 5 years of implementation of screening according to the budget impact guidelines [42].

### 2.8. Sensitivity Analyses

We performed one-way sensitivity analyses and probabilistic analyses (PSA) for all strategies. The one-way sensitivity analyses were performed to examine the effect of variation in parameters on the ICER and to reveal how a particular uncertainty affected the ICER. The parameters with the most pronounced influence on the ICER are shown in the form of a tornado diagram. The variation in the parameters was based on the 95% confidence interval. If the 95% confidence interval was unknown, we varied the parameter value between minus 20% and plus 20% to show the possible dependency of these parameters on the ICER. The results of this analysis are also represented in a tornado diagram.

In the PSA, 5000 simulations were performed. Variables and ranges are given in Table S2. For every simulation, the parameters were sampled from the 95% confidence interval of the distribution. If the 95% confidence interval was unknown, we varied the parameters between minus 20% and plus 20%. Prevalence, transition-probabilities and utilities values were sampled from a beta distribution. Cost parameters were described by a gamma distribution.

## 3. Results

The cost and QALYs results of the HCV screening strategies (i)–(iii), i.e., no screening, screening of migrants from HCV-endemic countries and screening of all migrants respectively, are presented in Table 3.

The incremental costs and QALYs from Table 3 are used to calculate the ICERs for the different strategies, as presented in Table 4. The ICER obtained for strategy (ii) screening of migrants from HCV-endemic countries versus strategy (i) no screening was €971 per QALY gained. The ICER of strategy (iii) screening of all migrants versus (ii) screening of migrants from HCV-endemic countries was €1005 per QALY gained. The relative cost-effectiveness ratio of the screening of all migrants over the no screening strategy was €982 per QALY gained.

### 3.1. Budget Impact of HCV Screening and Treatment

The budget impact concerns the costs of screening and treatment of diagnosed HCV patients in the first five years. As indicated in Table 3, there is a substantial difference in the budget impact of the strategies (ii) screening of migrants from HCV-endemic countries (€13,752,039) and (iii) screening of all migrants (€20,786,863). Indeed, implementation of HCV screening among just recently arrived migrants from HCV-endemic countries instead of recently arrived migrants from all Middle Eastern countries reduces the budget impact substantially.

### 3.2. Sensitivity Analyses

To explore the effect of uncertainty of different parameters, we performed both a one-way sensitivity analysis and a probabilistic sensitivity analysis, comparing strategy (ii) screening of migrants from HCV-endemic countries to (i) no screening, and strategy (iii) screening of all migrants to strategy (ii) screening of migrants from HCV-endemic countries. 

**Figure 2:**
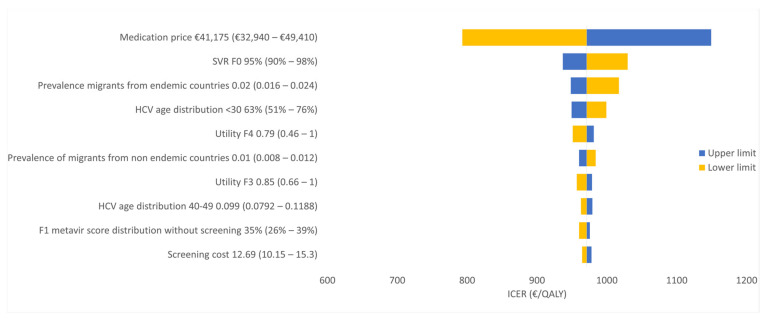
One-way sensitivity analysis for the (ii) screening of migrants from HCV-endemic countries strategy versus the (i) no screening strategy.

**Figure 3:**
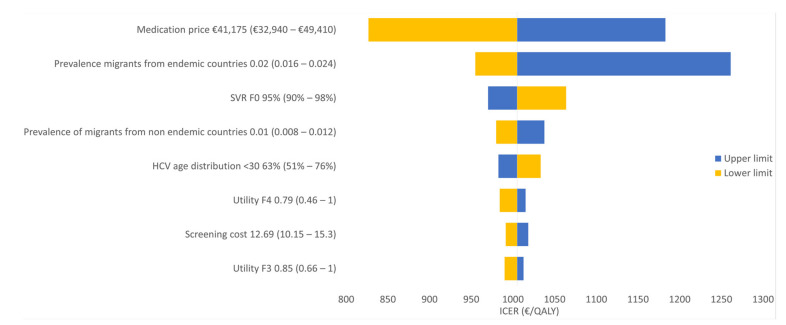
One-way sensitivity analysis for the (iii) HCV screening of all migrants strategy versus the (ii) screening of migrants from HCV-endemic countries strategy.

The one-way sensitivity analysis showed that the estimated medication price and estimated HCV prevalence had the most pronounced effect on the ICER (Figure 2 and Figure 3). Other parameters had a much smaller impact on the ICER.

**Figure 4:**
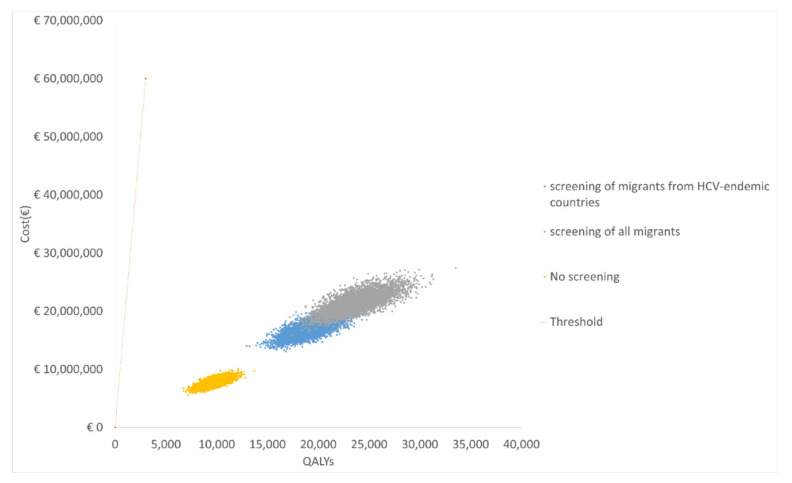
Cost-effectiveness plane.

**Figure 5:**
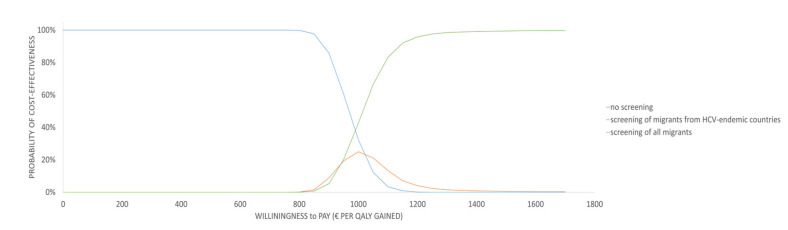
Cost-effectiveness acceptability curve.

The probabilistic analysis (PSA), shown in Figure 4, indicates that in all simulations, the ICERs of all considered strategies were well below the informal Dutch WTP threshold of €20,000 per QALY gained [22]. The cost-effectiveness acceptability curve, shown in Figure 5, indicates that a WTP of €1600 per QALY gained will result in almost 100% cost-effectiveness of strategy (iii) screening of all migrants. Transforming the cost-effectiveness acceptability curve to a cost-effectiveness acceptability frontier would only yield a probability trajectory for strategy (ii) screening of migrants from HCV-endemic countries between a WTP of €969 and €1003 gained (result not shown). Therefore, above WTP of €1003, screening of all migrants was more cost-effective than no screening and screening of migrants from HCV-endemic countries.

## 4. Discussion

In this study, we evaluated the cost-effectiveness of HCV screening and treatment strategies among recently arrived migrants in The Netherlands. Our evaluation showed that the strategy (ii) screening of migrants from HCV-endemic countries compared to (i) no screening, and the strategy (iii) screening of all migrants compared to (ii) screening of migrants from HCV-endemic countries are cost-effective. Indeed, the two strategies resulted in ICERs well below the informal Dutch and widely accepted WTP threshold of €20,000 per QALY gained. Our favorable cost-effectiveness results are driven by the early detection of HCV infection and the enhanced effectiveness of treatment if provided earlier in the process of infection progression, with corresponding decreases in HCV-complications and related costs and QALY gains. Notably, the costs of screening are relatively modest, in particular if compared with the subsequent costs of treatment. A final core assumption driving our results is that generally, all HCV-infected persons would need relatively expensive treatment, both in the presence as well as in the absence of screening. This seems a realistic assumption given that the new HCV treatments have evidenced effectiveness in all stages of infection, both early as well as late (although reduced in the latter, as mentioned above).

In an earlier study, Wong et al. determined the cost-effectiveness of implementation of HCV screening and subsequent treatment of all individuals living in Canada between 25 and 64 years of age with an estimated HCV prevalence of 0.5% and found an ICER of €34,783 per QALY gained [43]. However, the costs of DAA treatment in this study were substantially higher than the treatment costs used in our study and Wong et al. included all individuals living in Canada between 25 and 64 years of age [43]. Moreover, Wong et al. assumed that only patients with HCV genotype 1 infection will be offered DAA therapy instead of the, at that point, standard-of-care including interferon treatment. Urbanus et al. investigated the cost-effectiveness of HCV screening among pregnant women in The Netherlands and concluded that implementation of such a screening program in general is not cost-effective [4]. On the other hand, in the same study, HCV screening of pregnant first-generation non-Western women showed a modest cost-effective outcome, even though DAA treatment was not yet included [4]. A recent study of Suijkerbuijk et al., which did include DAA treatment of all chronic HCV patients after screening, indicates that HCV screening among migrants resulted an ICER ranging from €4962 per QALY gained for migrants originating from the Former Soviet Union (HCV prevalence 3.83%) and Vietnam (HCV prevalence 7.72%), to €9375 per QALY gained for Polish migrants (HCV prevalence 0.50%) [44]. Our results are comparable with the results of the study of Suijkerbuijk et al., in that HCV prevalence has a substantial effect on the ICER.

The ICERs found in our study were mostly affected by the medication price and the estimated prevalence of HCV and percentage of patients who reach SVR at F0, as indicated by the one-way sensitivity analyses (Figure 2 and Figure 3). Yet, both considered strategies remained cost-effective over the whole range of the medication price and prevalence evaluated in the sensitivity analyses. A break-even analysis—for example, on prevalence—appeared to be uninformative as our cost-effectiveness results were dominated by the treatment costs for detected cases on the one hand and heavily discounted future savings on HCV complications on the other hand. Therefore, for all realistic options investigated, incremental costs always remained positive in combination with QALY gains. The probabilistic analysis showed that the uncertainty of prevalence, cost and utility parameters in this model did not affect the ICER substantially. The cost-effectiveness acceptability curve showed that our strategy (iii) screening of all migrants is almost 100% cost-effective using a WTP of €1600 per QALY. Screening of migrants from HCV-endemic countries had low probability to be cost-effective. Therefore, this strategy does not represent an optimal option.

As indicated above, both screening strategies are cost-effective, however the budget impact in this study reveals a substantial difference between the different strategies. Indeed, implementation of strategy (ii) screening of migrants from HCV-endemic countries compared to (i) no screening resulted in a budget impact of €13,752,039, whereas strategy (iii) screening of all migrants compared to (i) no screening resulted in a budget impact of €20,786,683. The main reason is that the cohort of migrants from HCV-endemic countries is smaller than the cohort of all migrants. This, therefore, may favor decision-makers to screen small high-risk groups only. Notably, expiring patents on DAA treatment will result in lower costs of HCV treatment in the future.

Even though we have conducted our study for The Netherlands, the results may be applicable to other developed countries and could be used as an indicator to implement HCV screening programs. However, the budget impact may vary, because the number of migrants may differ substantially among different countries. Nevertheless, countries with comparable numbers of migrants, screening and treatment prices to those in The Netherlands can expect approximately the same budget impact.

There are a few limitations to our study that should be noted. Firstly, costs of DAA treatment are not publicly known as these are directly negotiated between the drug manufacturer and the Ministry of Health in The Netherlands. To address this limitation, we used sensitivity analyses. The one-way sensitivity analysis showed that the price of the treatment has a substantial influence on the ICER. Transparency about the treatment price would, therefore, help to determine the exact ICER of screening and treatment. Secondly, we did not include retreatment in our analysis and the long-term effects of DAA treatment are unknown. In practice, there will be retreatment after failed treatment. This could result in a higher ICER of HCV screening and treatment. However, DAA treatment has a high efficacy, which implies that treatment failure will be limited. Thirdly, the risk of re-infection and transmission is not covered in this study and possible overlap between different HCV risk groups was disregarded. However, we expect that the chance of re-infection and transmission in this specific group is low. The HCV patients in this group are mostly infected due to poor healthcare practices in their home countries. In general, HCV infection in this group is not caused by drug abuse or by sexual transmission among men-who-have-sex-with-men (MSM). HCV prevalence could be affected to some extent by overlap between different risk groups, for example, recently arrived MSM and migrants. Fourthly, the HCV screening program has not been investigated as a randomized control trial (RCT). In the absence of such an RCT on migrants, we used modeling to estimate the value of an HCV screening program in this target group. Our modeling study might be biased compared to an economic study based on an RCT or even real-world data, with the latter two settings potentially showing difficulties in including vulnerable parts of populations potentially at more than average risk. Our modeling study assumes all relevant populations to be included, inclusive of the most vulnerable groups with potentially higher risks for HCV. Given the dominant part of the treatment costs in our analysis, we would not expect this to highly influence cost-effectiveness though.

## 5. Conclusions

Our analysis shows that HCV screening of migrants from HCV-endemic countries is a highly cost-effective intervention in The Netherlands. From our economic perspective and incremental analysis, it followed that screening of all migrants is highly cost-effective as well. However, the difference in budget impact between both screening strategies may also be taken into account by decision makers.

## Figures and Tables

**Table 1:** Age distribution with corresponding percentage of total recently arrived migrants in the Netherlands in 2017.

Migrants Age	Percentage [20]
0–3	6.8
4–11	12.3
12–17	10.7
18–29	33.8
30–39	20.2
40–49	9.9
50–59	4.1
60>	2.2

**Table 2:** Country of origin with corresponding percentage of total recently arrived migrants in the Netherlands in 2017 and HCV prevalence.

Country of Origin	Percentage [11]	HCV Prevalence [12,13,14,15]
Syria	51%	1%
Eritrea	13%	1.9%
Iraq	4.3%	3.2%
Morocco	2.6%	7.7%
Algeria	2.4%	1.8%
Weighted Average		1.5%

**Table 3:** The total costs and quality-adjusted life-years (QALYs) of each HCV screening strategy among migrants.

Scenario	Cohort	Screening Costs	HCV Treatment Costs	HCV Follow up Costs	HCV Complications Costs	Total Costs Discounted	Total Discounted Costs of Both Cohorts	QALYs per Cohort	QALYs per Cohort Discounted	Total Discounted QALYs of Both Cohorts
(i) No screening	HCV-endemic countries	€0	€13,243,572	€1,872,576	€4,251,432	€5,138,945	€7,708,418	12,645	6446	9669
	Non HCV-endemic countries	€0	€6,621,786	€936,288	€1,657,572	€2,569,473		6322	3223	
(ii) Screening of migrants from HCV-endemic countries	HCV-endemic countries	€496,250	€13,214,500	€103,286	€2,461,026	€14,295,753	€16,865,226	23,063	15,875	19,098
	Non HCV-endemic countries	€0	€6,621,786	€936,288	€1,657,572	€2,569,473		6322	3223	
(iii) Screening of all migrants	HCV-endemic countries	€496,250	€13,214,500	€103,286	€2,461,026	€14,295,753	€21,602,254	23,063	15,875	23,812
	Non HCV-endemic countries	€406,750	€6,607,250	€51,643	€1,389,138	€7,306,501		11,532	7937	

**Table 4:** Incremental cost-effectiveness of different strategies of HCV screening and treatment among migrants in The Netherlands.

Scenario	Total Cost	Incremental Cost	QALYs	QALYs Gained	ICER
(i) No screening	€7,708,418	-	9669	-	-
(ii) Screening of migrants from HCV-endemic countries	€16,865,226	€9,156,808	19,098	9429	€971
(iii) Screening of all migrants	€21,602,254	€4,737,029	23,812	4714	€1005

QALY, quality-adjusted life-years; ICER, incremental cost-effectiveness ratio.

## Supplementary Materials

The following are available online at https://www.mdpi.com/1660-4601/17/17/6091/s1, Table S1: parameters of Markov model hepatitis C, Table S2: PSA parameters variation.
