# Supplementary material for: The Cost-Effectiveness of Hepatitis C Virus Screening Strategies among Recently Arrived Migrants in the Netherlands
Source: Int J Environ Res Public Health. 2020 Aug 21;17(17):6091. doi: 10.3390/ijerph17176091 (PMC7503411; doi:10.3390/ijerph17176091)
Supplement: Supplementary file 1 [file ijerph-17-06091-s001.zip › ijerph-886761-suppl/Appendix S1.docx]

**S1 Appendix: parameters of Markov model hepatitis C**

| **Transition state** | | |
| --- | --- | --- |
| **From** | **To** | **Probability** |
| Treatment | SVR F0 | 0.95 (1) |
|  | SVR F1 | 0.95 (1) |
|  | SVR F2 | 0.95 (1) |
|  | SVR F3 | 0.95 (1) |
|  | SVR F4 | 0.9 (1) |
| Non cirrhotic F0-F3 | Cirrhosis F4 | 0.073 (2) |
| Cirrhosis F4 | DC | 0.039 (2) |
|  | HCC | 0.037 (2) |
|  | Death | 0.053 (2) |
| DC | HCC | 0.037 (2) |
|  | LT | 0.03 (2) |
|  | Death | 0.13 (3) |
| HCC | Death | 0.43 (4,5) |
| LT | Post LT1 | 0.79 (4,5) |
|  | Death | 0.21 (4,5) |
| Post LT1 | Post LT+ | 0.943 (4,5) |
|  | Death | 0.057 (4,5) |
| POST LT+ | Death | 0.02 (4,5) |
| F0 | F1 | 0.117 (6) |
| F1 | F2 | 0.085 (6) |
| F2 | F3 | 0.121 (6) |
| F3 | F4 | 0.115 (6) |
| SVR | F0 | 0.05 (1) |
| SVR | F1 | 0.05 (1) |
| SVR | F2 | 0.05 (1) |
| SVR | F3 | 0.05 (1) |
| SVR | F4 | 0.1 (1) |
| SVR F4 | DC | 0.004 (7) |
| SVR F4 | HCC | 0.005 (7) |

***METAVIR score: F0,F1,F2,F3,F4. SVR: Sustained Virologic response. HCC: hepatocellular cancer. DC: Decompensated cirhossis.LT: liver transplantation. Post LT1: post-liver transplantation care year 1. Post LT1+: post-liver transplantation care after year 1***

| **Annual healthcare costs** | **Costs** |  |
| --- | --- | --- |
| SVR F0-F3 | € 426 (8) |  |
| SVR F4 | € 673 (8) |  |
| F0-F3 | € 286 (8) |  |
| F4 | € 821 (9) |  |
| DC | € 27,921 (10) |  |
| HCC | € 21,054 (9) |  |
| LT | €143,226 (9) |  |
| Post LT1 | € 20,714 (9) |  |
| Post LT+ | € 20,714 (8) |  |
|  |  |  |
| **Average treatment costs (only once)** | **HCV treatment** | **Medical costs** |
| treatment F0-F4 (12W) | € 24,750 (11,12) | € 1,679 (8) |
|  |  |  |
| **Costs screening** | **Cost** |  |
| serological test | €12,69 (13) |  |
| PCR test | €122 (13) |  |
| Fibroscan | €236 (13) |  |
| Monitoring | €100 (14) |  |

***METAVIR score: F0,F1,F2,F3,F4. SVR: Sustained Virologic response. HCC: hepatocellular cancer. DC: Decompensated cirhossis.LT: liver transplantation. Post LT1: post-liver transplantation care year 1. Post LT1+: post-liver transplantation care after year 1***

***METAVIR score: F0,F1,F2,F3,F4. HCC: hepatocellular cancer. DC: Decompensated cirhossis.LT: liver transplantation. Post LT1: post-liver transplantation care year 1. Post LT1+: post-liver transplantation care after year 1***

| **Distribution of HCV by age among migrants** |  |
| --- | --- |
| <30 | 0.636  **^assumed^** |
| 30-39 | 0.202  **^assumed^** |
| 40-49 | 0.099  **^assumed^** |
| 50-59 | 0.041  **^assumed^** |
| 60-69 | 0.022  **^assumed^** |

| **Prevalence** |  |
| --- | --- |
| HCV prevalence of migrants from Middle-Eastern countries | 0.015 (15–17) |
| HCV prevalence of migrants from HCV endemic countries | 0.02 **^assumed^** |

| **Baseline utilities** |  |
| --- | --- |
| F0 | 0.97 (18,19) |
| F1-F2 | 0.95 (18,19) |
| F3 | 0.85 (18,19) |
| F4 | 0.79 (18,19) |
| DC | 0.72 (18,19) |
| HCC | 0.72 (18,19) |
| LT | 0.5 (18,19) |
| PostLT | 0.7 (18,19) |
| Post LT + | 0.825 (18,19) |

| **Distribution of Metavir stage with and without screening** |  |
| --- | --- |
| Metavir score screening F0 | 0.89 **^assumed^** |
| Metavir score screening F1 | 0.08  **^assumed^** |
| Metavir score screening F2 | 0.02  **^assumed^** |
| Metavir score screening F3 | 0.008  **^assumed^** |
| Metavir score screening F4 | 0.002  **^assumed^** |
| Metavir score No Screening F0 | 0.17 (20) |
| Metavir score No Screening F1 | 0.35 (20) |
| Metavir score No Screening F2 | 0.22 (20) |
| Metavir score No Screening F3 | 0.14 (20) |
| Metavir score No Screening F4 | 0.12 (20) |

**References**

1. WHO | Guidelines for the screening, care and treatment of persons with chronic hepatitis C infection. WHO. 2016;

2. Younossi ZM, Singer ME, McHutchison JG, Shermock KM. Cost effectiveness of interferon ?2b combined with ribavirin for the treatment of chronic hepatitis C. Hepatology. 1999 Nov;30(5):1318–24.

3. Salomon JA. Cost-effectiveness of Treatment for Chronic Hepatitis C Infection in an Evolving Patient Population. JAMA. 2003 Jul 9;290(2):228.

4. Interferon alpha (pegylated and non-pegylated) and ribavirin for the treatment of mild chronic hepatitis C: a systematic review and economic evalua... - PubMed - NCBI.

5. Plunkett BA, Grobman WA. Routine hepatitis C virus screening in pregnancy: A cost-effectiveness analysis. Am J Obstet Gynecol. 2005 Apr;192(4):1153–61.

6. Thein H-H, Yi Q, Dore GJ, Krahn MD. Estimation of stage-specific fibrosis progression rates in chronic hepatitis C virus infection: A meta-analysis and meta-regression. Hepatology. 2008 Aug 1;48(2):418–31.

7. Townsend R, McEwan P, Kim R, Yuan Y. Structural Frameworks and Key Model Parameters in Cost-Effectiveness Analyses for Current and Future Treatments of Chronic Hepatitis C. Value Heal. 2011 Dec;14(8):1068–77.

8. Richtlijn voor het uitvoeren van economische evaluaties in de gezondheidszorg | Publicatie | Zorginstituut Nederland [Internet]. [cited 2017 Sep 20]. Available from: https://www.zorginstituutnederland.nl/over-ons/publicaties/publicatie/2016/02/29/richtlijn-voor-het-uitvoeren-van-economische-evaluaties-in-de-gezondheidszorg

9. Siebert U, Sroczynski G, Rossol S, Wasem J, Ravens-Sieberer U, Kurth BM, et al. Cost effectiveness of peginterferon alpha-2b plus ribavirin versus interferon alpha-2b plus ribavirin for initial treatment of chronic hepatitis C. Gut. 2003 Mar;52(3):425–32.

10. Singer ME, Younossi ZM. Cost effectiveness of screening for hepatitis C virus in asymptomatic, average-risk adults. Am J Med. 2001 Dec;111(8):614–21.

11. HCV Richtsnoer - Home - Hepatitis C [Internet]. [cited 2017 Sep 10]. Available from: https://hcvrichtsnoer.nl/

12. Welkom bij Medicijnkosten [Internet]. [cited 2017 Sep 12]. Available from: https://www.medicijnkosten.nl/

13. Urbanus AT, van Keep M, Matser AA, Rozenbaum MH, Weegink CJ, van den Hoek A, et al. Is Adding HCV Screening to the Antenatal National Screening Program in Amsterdam, The Netherlands, Cost-Effective? Jhaveri R, editor. PLoS One. 2013 Aug 12;8(8):e70319.

14. Afdhal NH. Fibroscan (transient elastography) for the measurement of liver fibrosis. Gastroenterol Hepatol (N Y). 2012 Sep;8(9):605–7.

15. Daw MA, Dau AA. Hepatitis C Virus in Arab World: A State of Concern. Sci World J. 2012 May 2;2012:1–12.

16. Gower E, Estes C, Blach S, Razavi-Shearer K, Razavi H. Global epidemiology and genotype distribution of the hepatitis C virus infection. J Hepatol. 2014 Nov;61(1):S45–57.

17. Mohd Hanafiah K, Groeger J, Flaxman AD, Wiersma ST. Global epidemiology of hepatitis C virus infection: New estimates of age-specific antibody to HCV seroprevalence. Hepatology. 2013 Apr;57(4):1333–42.

18. Liu S, Cipriano LE, Holodniy M, Owens DK, Goldhaber-Fiebert JD. New Protease Inhibitors for the Treatment of Chronic Hepatitis C. Ann Intern Med. 2012 Feb 21;156(4):279.

19. McLernon DJ, Dillon J, Donnan PT. Systematic Review: Health-State Utilities in Liver Disease: A Systematic Review. Med Decis Mak. 2008 Jul 18;28(4):582–92.

20. Chahal HS, Marseille EA, Tice JA, Pearson SD, Ollendorf DA, Fox RK, et al. Cost-effectiveness of Early Treatment of Hepatitis C Virus Genotype 1 by Stage of Liver Fibrosis in a US Treatment-Naive Population. JAMA Intern Med. 2016;176(1):65.
