# Supplementary material for: The Cost-Effectiveness of Hepatitis C Virus Screening Strategies among Recently Arrived Migrants in the Netherlands
Source: Int J Environ Res Public Health. 2020 Aug 21;17(17):6091. doi: 10.3390/ijerph17176091 (PMC7503411; doi:10.3390/ijerph17176091)
Supplement: Supplementary file 1 [file ijerph-17-06091-s001.zip › ijerph-886761-suppl/Appendix S2.docx]

| **S2 Appendix: PSA parameters variation** | | | | | |
| --- | --- | --- | --- | --- | --- |
| ***Parameter*** | | ***Value*** | ***Low*** | ***High*** | ***Distribution*** |
| **Prevalence** | |  |  |  |  |
| HCV prevalence of migrants from Middle-Eastern countries | | 0.015 (1–3) | 0.007 | 0.023 | Beta |
| HCV prevalence of migrants from HCV endemic countries | | 0.02 **^assumed^** | 0.015 | 0.025 | Beta |
| **Distribution of Metavir stage with and without screening** | |  |  |  |  |
| Metavir score screening F0 | | 0.89 **^assumed^** | 0.801 | 0.979 | Beta |
| Metavir score screening F1 | | 0.08  **^assumed^** | 0.072 | 0.088 | Beta |
| Metavir score screening F2 | | 0.02  **^assumed^** | 0.018 | 0.022 | Beta |
| Metavir score screening F3 | | 0.008  **^assumed^** | 0.0072 | 0.0088 | Beta |
| Metavir score screening F4 | | 0.002  **^assumed^** | 0.0018 | 0.0022 | Beta |
| Metavir score No Screening F0 | | 0.17 (4) | 0.14 | 0.19 | Beta |
| Metavir score No Screening F1 | | 0.35 (4) | 0.26 | 0.39 | Beta |
| Metavir score No Screening F2 | | 0.22 (4) | 0.18 | 0.24 | Beta |
| Metavir score No Screening F3 | | 0.14 (4) | 0.12 | 0.15 | Beta |
| Metavir score No Screening F4 | | 0.12 (4) | 0.11 | 0.13 | Beta |
| **Costs** | |  |  |  |  |
| Serological test | | €12.69 (5) | 11.421 | 13.959 | Gamma |
| PCR test | | €122 (6) | 109.8 | 134.2 | Gamma |
| Fibroscan | | €236 (6) | 212.4 | 259.6 | Gamma |
| Monitoring | | €100 (7) | 90 | 110 | Gamma |
| SVR F0-F3 | | € 426 (8) | 383.4 | 468.6 | Gamma |
| SVR F4 | | € 673 (8) | 605.7 | 740.3 | Gamma |
| F4 | | € 821 (8) | 738.9 | 903.1 | Gamma |
| F4 | | € 821 (8) | 738.9 | 903.1 | Gamma |
| DCC | | € 27,921 (8) | 25128.9 | 30713.1 | Gamma |
| HCC | | € 21,054 (8) | 18948.6 | 23159.4 | Gamma |
| LT | | € 143,226 (8) | 128903.4 | 157548.6 | Gamma |
| Post LT | | € 20,714 (8) | 18642.6 | 22785.4 | Gamma |
| Post LT 2 | | € 20,714 (8) | 18642.6 | 22785.4 | Gamma |
| **Baseline utilities** | |  |  |  |  |
| F0 | | 0.97 (9,10) | 0.873 | 1 | Beta |
| F1-F2 | | 0.95 (9,10) | 0.7 | 1 | Beta |
| F3 | | 0.85 (9,10) | 0.66 | 1 | Beta |
| F4 | | 0.79 (9,10) | 0.46 | 1 | Beta |
| DC | | 0.72 (9,10) | 0.26 | 0.91 | Beta |
| HCC | | 0.72 (9,10) | 0.15 | 0.95 | Beta |
| LT | | 0.5 (9,10) | 0.2 | 0.8 | Beta |
| PostLT | | 0.7 (9,10) | 0.2 | 0.8 | Beta |
| Post LT + | | 0.825 (9,10) | 0.64 | 1 | Beta |
| **Distribution of HCV by age among migrants** | |  |  |  |  |
| <30 | | 0.636  **^assumed^** | 0.5724 | 0.6996 | Beta |
| 30-39 | | 0.202  **^assumed^** | 0.1818 | 0.2222 | Beta |
| 40-49 | | 0.099  **^assumed^** | 0.0891 | 0.1089 | Beta |
| 50-59 | | 0.041  **^assumed^** | 0.0369 | 0.0451 | Beta |
| 60-69 | | 0.022  **^assumed^** | 0.0198 | 0.0242 | Beta |
| **Transition state** | |  |  |  |  |
| From | to | Transition probabilities |  |  |  |
| Treatment | SVR F0 | 0.95 (11) | 0.9 | 0.98 | Beta |
|  | SVR F1 | 0.95 (11) | 0.9 | 0.98 | Beta |
|  | SVR F2 | 0.95 (11) | 0.9 | 0.98 | Beta |
|  | SVR F3 | 0.95 (11) | 0.9 | 0.98 | Beta |
|  | SVR F4 | 0.9 (11) | 0.85 | 0.95 | Beta |
| F0-F3 | F4 | 0.073 (12) | 0.01 | 0.232 | Beta |
| F4 | DCC | 0.039 (12) | 0.02 | 0.083 | Beta |
|  | HCC | 0.037 (12) | 0.01 | 0.044 | Beta |
|  | Death | 0.053 (12) | 0.04 | 0.06 | Beta |
| DCC | HCC | 0.037 (12) | 0.01 | 0.044 | Beta |
|  | LT | 0.03 (12) | 0.01 | 0.062 | Beta |
|  | Death | 0.13 (13) | 0.065 | 0.193 | Beta |
| HCC | Death | 0.43 (14,15) | 0.33 | 0.86 | Beta |
| LT | Post LT1 | 0.79 (14,15) | 1 | 1 |  |
|  | Death | 0.21 (14,15) | 0.06 | 0.42 | Beta |
| Post LT1 | Post LT+ | 0.943 (14,15) | 1 | 1 |  |
|  | Death | 0.057 (14,15) | 0.024 | 0.11 | Beta |
| POST LT+ | Death | 0.02 (14,15) | 0.012 | 0.042 | Beta |
| F0 | F1 | 0.117 (16) | 0.107 | 0.127 | Beta |
| F1 | F2 | 0.085 (16) | 0.078 | 0.093 | Beta |
| F2 | F3 | 0.121 (16) | 0.112 | 0.13 | Beta |
| F3 | F4 | 0.115 (16) | 0.107 | 0.123 | Beta |

***METAVIR score: F0.F1.F2.F3.F4. SVR: Sustained Virologic response. HCC: hepatocellular cancer. DC: Decompensated cirhossis.LT: liver transplantation. LRD: Liver related death***

**References**

1. Daw MA, Dau AA. Hepatitis C Virus in Arab World: A State of Concern. Sci World J. 2012 May 2;2012:1–12.

2. Gower E, Estes C, Blach S, Razavi-Shearer K, Razavi H. Global epidemiology and genotype distribution of the hepatitis C virus infection. J Hepatol. 2014 Nov;61(1):S45–57.

3. Mohd Hanafiah K, Groeger J, Flaxman AD, Wiersma ST. Global epidemiology of hepatitis C virus infection: New estimates of age-specific antibody to HCV seroprevalence. Hepatology. 2013 Apr;57(4):1333–42.

4. Chahal HS, Marseille EA, Tice JA, Pearson SD, Ollendorf DA, Fox RK, et al. Cost-effectiveness of Early Treatment of Hepatitis C Virus Genotype 1 by Stage of Liver Fibrosis in a US Treatment-Naive Population. JAMA Intern Med. 2016;176(1):65.

5. Urbanus AT, van Keep M, Matser AA, Rozenbaum MH, Weegink CJ, van den Hoek A, et al. Is Adding HCV Screening to the Antenatal National Screening Program in Amsterdam, The Netherlands, Cost-Effective? Jhaveri R, editor. PLoS One. 2013 Aug 12;8(8):e70319.

6. Liu S, Schwarzinger M, Carrat F, Goldhaber-Fiebert JD. Cost Effectiveness of Fibrosis Assessment Prior to Treatment for Chronic Hepatitis C Patients. Jhaveri R, editor. PLoS One. 2011 Dec 2;6(12):e26783.

7. Afdhal NH. Fibroscan (transient elastography) for the measurement of liver fibrosis. Gastroenterol Hepatol (N Y). 2012 Sep;8(9):605–7.

8. Richtlijn voor het uitvoeren van economische evaluaties in de gezondheidszorg | Publicatie | Zorginstituut Nederland [Internet]. [cited 2017 Sep 20]. Available from: https://www.zorginstituutnederland.nl/over-ons/publicaties/publicatie/2016/02/29/richtlijn-voor-het-uitvoeren-van-economische-evaluaties-in-de-gezondheidszorg

9. Liu S, Cipriano LE, Holodniy M, Owens DK, Goldhaber-Fiebert JD. New Protease Inhibitors for the Treatment of Chronic Hepatitis C. Ann Intern Med. 2012 Feb 21;156(4):279.

10. McLernon DJ, Dillon J, Donnan PT. Systematic Review: Health-State Utilities in Liver Disease: A Systematic Review. Med Decis Mak. 2008 Jul 18;28(4):582–92.

11. WHO | Guidelines for the screening, care and treatment of persons with chronic hepatitis C infection. WHO. 2016;

12. Younossi ZM, Singer ME, McHutchison JG, Shermock KM. Cost effectiveness of interferon ?2b combined with ribavirin for the treatment of chronic hepatitis C. Hepatology. 1999 Nov;30(5):1318–24.

13. Salomon JA. Cost-effectiveness of Treatment for Chronic Hepatitis C Infection in an Evolving Patient Population. JAMA. 2003 Jul 9;290(2):228.

14. Interferon alpha (pegylated and non-pegylated) and ribavirin for the treatment of mild chronic hepatitis C: a systematic review and economic evalua... - PubMed - NCBI.

15. Plunkett BA, Grobman WA. Routine hepatitis C virus screening in pregnancy: A cost-effectiveness analysis. Am J Obstet Gynecol. 2005 Apr;192(4):1153–61.

16. Thein H-H, Yi Q, Dore GJ, Krahn MD. Estimation of stage-specific fibrosis progression rates in chronic hepatitis C virus infection: A meta-analysis and meta-regression. Hepatology. 2008 Aug 1;48(2):418–31.
